# Patterns in the prevalence and wealth-based inequality of cervical cancer screening in India

**DOI:** 10.1186/s12905-023-02504-y

**Published:** 2023-06-26

**Authors:** M. R. Muthuramalingam, V. R. Muraleedharan

**Affiliations:** Department of Humanities and Social Sciences, Indian Institute of Technology – Madras, Chennai, India

**Keywords:** Cervical cancer, Screening prevalence, Inequality, Health Insurance

## Abstract

**Background:**

Cervical cancer is the second leading cause of deaths due to cancer among women in India. This study assesses the prevalence of cervical cancer screening among women in the 30 to 49 years age-group and its relation to demographic, social and economic factors. The equity in the prevalence of screening is studied with respect to the women’s household wealth.

**Methods:**

Data from the fifth National Family Health Survey are analyzed. The adjusted odds ratio is used to assess the prevalence of screening. The Concentration Index (CIX) and the Slope Index of Inequality (SII) are analyzed to assess the inequality.

**Results:**

The average national prevalence of cervical cancer screening is found to be 1.97% (95% C.I, 1.8–2.1), ranging from 0.2% in West Bengal and Assam to 10.1% in Tamil Nadu. Screening is significantly more prevalent among the following demographics: educated, higher age group, Christian, scheduled caste, Government health insurance coverage, and high household wealth. Significantly lower prevalence is found among Muslim women, women from scheduled tribes, general category castes, non-Government health insurance coverage, high parity, and those who use oral contraceptive pills and tobacco. Marital status, place of residence, age at first sexual activity, and IUD usage are not significant influencers. At the national level, CIX (0.22 (95% C.I, 0.20–0.24)) and SII (0.018 (95% C.I, 0.015–0.020)) indicate significantly higher prevalence of screening among women from the wealthier quintiles. Significantly higher screening prevalence among wealthier quintiles in the North-East (0.1), West (0.21) and Southern (0.05) regions and among the poor quintiles in the Central (-0.05) region. Equiplot analysis shows a “top inequality pattern” in the North, North-East and Eastern regions, with overall low performance where the rich alone manage to avail screening. The Southern region exhibits an overall progress in screening prevalence with the exception of the poorest quintile, which is left behind. Pro-poor inequality exists in the Central region, with significantly higher prevalence of screening among poor.

**Conclusion:**

The prevalence of cervical cancer screening is very low (2%) in India. Cervical cancer screening is substantially higher among women with education and Government Health insurance coverage. Wealth-based inequality exists in the prevalence of cervical cancer screening and the prevalence is concentrated among the women from wealthier quintiles.

## Introduction

### The burden of cervical cancer

Cervical cancer (cancer of the uterine cervix) was the fourth most common cancer and the fourth leading cause of cancer deaths in women worldwide during the year 2020, with an estimated 6,04,127 new cases and 3,41,831 deaths [1]. In India, cervical cancer is the second most common cancer among women with 1,23,907 new cases, and the second leading cause of cancer deaths in women with 77,348 estimated deaths during 2020. In the same year, India accounted for 21% of new cases of cervical cancer and 23% of deaths due to cervical cancer in the world. This makes India the country with the highest number of new cases of cervical cancer and the highest number of deaths due to cervical cancer, surpassing China, which was leading in incidence of cervical cancer until 2018 [2].

The peak age of incidence for cervical cancer in India is 50–59 years, compared to 35–44 years in developed countries [3]. This high age group is partially attributable to the considerable proportion of women in India who are diagnosed with cervical cancer when they are already in the advanced and late stages of the disease: 32.8% of patients have localized disease and for 67.2% of patients, the disease has spread beyond the uterine cervix at the time of initial diagnosis [4, 5]. This makes treatment costly and provides a poor prognosis, resulting in higher mortality rates. The 5-year relative survival rate of cervical cancer (all stages combined for all people) is 46% in India compared to 66% in the United States [3, 6]. With the current population growth rate, the absolute number of new cases of cervical cancer for all ages in India in 2040 is estimated to be 1,91,347 – an increase of 54% over the number of new cases reported during 2020. The corresponding mortalities due to cervical cancer in India in 2040 are estimated to be 1,24,677 – an increase of 61% over the estimated number of deaths due to cervical cancer in 2020 [7, 8].

### Cervical cancer screening

The importance of screening for cervical cancer cannot be overemphasized, because diagnosis at the earliest stage of disease is the key to achieving a complete cure without recurrence. Cervical Intraepithelial Neoplasia (CIN) is the precancerous lesion that could lead to cervical cancer. CIN is graded into Low grade Squamous Intraepithelial Lesion (LSIL) also called CIN I and High grade Squamous Intraepithelial Lesion (HSIL). HSIL is subdivided into CIN II and CIN III [9].

- CIN II: Moderate cervical dysplasia; high rate of regression to normal.
- CIN III: Marked full thickness atypia and loss of maturation; carries highest risk of progression to invasive squamous cell carcinoma.

A patient with lesions diagnosed and treated at stage CIN III is at significantly higher hazard risk (4.88) for persistence / recurrence than a patient with lesions that are diagnosed and treated during CIN II [10]. Early-stage cervical cancer is amenable to treatment, resulting in 95% disease-free survival and 98% overall survival at ten years [11].

In India, the prevalence of cervical cancer screening was reported as 29.8% among women aged 30–49 years and 22.3% among women aged 15–49 years, as per the fourth round of the National Family Health Survey (NFHS-4) conducted during the year 2015–16 [12, 13]. NFHS-4 collected responses to the question “Have you ever had cervix examination?” from the participant women to assess the prevalence of cervical cancer screening. Responses from the 6,99,686 women aged 15–49 years who participated in the survey revealed that 22.3% of them have had a cervix examination [12]. The fifth round of NFHS, conducted in 2019–21, rephrased the question more specifically as “Have you ever undergone a screening test for cervical cancer?”, which is more specific to cervical cancer, unlike the non-specific question posed during the previous round. Responses from 7,65,805 women aged 15–49 years (during NFHS-5) showed that 1.2% of them have undergone a screening test for cervical cancer [14]. This is far below the prevalence rate based on the NFHS-4 (22.3%), which is primarily due to the formulation of the question.

Considering the marked difference in the prevalence of screening for cervical cancer between NFHS-4 and NFHS-5 and the reason that NFHS-5 is based on the response to a more specific question on cervical cancer screening, the baseline for prevalence needs to be reset according to the NFHS-5 data. Our study, therefore, analyzes the baseline characteristics of cervical cancer screening in India based on the NFHS-5 data. We consider it inappropriate to analyze the prevalence trends over time, as the data from the previous rounds of the NFHS are not comparable with NFHS-5 with respect to the question on the prevalence of cervical cancer screening.

This paper is organized as follows:

- “Introduction” section presents the burden of cervical cancer and the need for resetting the baseline for cervical cancer screening prevalence.
- “Methods” section describes the methodology of the study and the indices used to assess the data.
- “Results” section elaborates the results of the analysis – the socio-demographic profile, the prevalence of cervical cancer screening, and the wealth-based inequality associated with it.
- “Discussion” section discusses the results of the study.

## Methods

### Database

The fifth round of the National Family Health Survey was conducted between June 2019 and April 2021, covering all the 28 states and eight union territories of India, comprising 707 districts. This survey, organized by the Ministry of Health and Family Welfare, gathered information from 6,36,699 households, 7,24,115 women, and 1,01,839 men. The sample is a stratified two-stage sample derived from the 2011 census representative at the national, state/union territory, and district level. A detailed description of the sampling design is presented in the NFHS-5 national report [14]. No ethical clearance is required, since this study involves the secondary analysis of the NFHS survey dataset, which is freely downloadable following a successful registration in the Demographic and Health Survey program. The data does not contain respondents’ names or any other identifiers.

### Data and variables

The NFHS-5 questionnaire contains information from women between 15–49 years of age who were identified as eligible for the survey. From this dataset, the data pertaining to the women in the age group of 30–49 years who answered the question “Have you ever undergone a screening test for cervical cancer?” were filtered and analyzed. The dependent variable was the response to the above question. The independent variables were socio-demographic factors – age in five-year groups, highest educational level, ever been married or in union, religion, caste, household wealth index, type of place of residence (rural vs urban), health insurance coverage (government vs non-government), occupation, partner’s education, region of residence (at the sub-national level – North, Central & others) – and the risk factors for cervical cancer – age at first sexual activity, number of child births, have used IUDs (intrauterine contraceptive devices), have used OCPs (oral contraceptive pills), tobacco use, and history of STI (Sexually Transmitted Infections).

### Analysis

Analysis was carried out using the statistical software STATA 15.1 (StataCorp LLC, Texas, USA). Sampling weights, stratification and clustering were accounted for in the analysis and datasets were declared as survey-type using the “svyset” command. Proportion, unweighted and weighted prevalence, and unadjusted and adjusted odds ratios with a 95% confidence interval were analyzed for the prevalence of cervical cancer screening. The national and sub-national prevalence of screening for cervical cancer among women in the 30-to-49-year age group and their relation to demographic, social and economic factors were analyzed. A collinearity check was performed to avoid multicollinearity. A test for goodness of fit was also performed to check whether the sample data fit an expected set of data from a population with normal distribution. The variable inflation factor (VIF) and tolerance were also checked. The adjusted odds ratio was calculated to compare the prevalence of screening with one predictor after adjusting for the other predictors.

The Concentration Index and the Slope Index of Inequality were analyzed to assess the inequality in the prevalence of cervical cancer screening with respect to the household wealth of the respondents. The data related to the quintile-wise wealth inequality of screening prevalence was plotted using the equiplot creator to study the impact of wealth inequality. The district- as well as state-level weighted prevalence was plotted in graphs to create maps. The indices of inequality and the equiplot are discussed in the results section.

## Results

### Socio-demographic profile

The NFHS-5 questionnaire collected information from 7,47,176 women between the ages of 15 and 49 who were identified as eligible for the survey. Among them, 7,24,115 women completed the questionnaire with a response rate of 96.9.%. Out of these responses, 3,57,353 were from the 30–49 years age group. The 30–34 age group constituted 27.5% and the 35–39 age group had 26.7% of the participants; the 40–44- and 45–49-years’ groups constituted 22.4% and 23.4%, respectively. Women with no formal education comprised 35.4% of respondents whereas 15.5% had primary education (5 years of schooling), 38.7% had completed secondary education (10 years of schooling), and 10.4% higher education (10 + years of schooling). A majority of the women (91.0%) were currently married, 7.4% were formerly married, and 1.6% were never married. 82.3% of participants were Hindu by religion, and 12.1% and 2.6% were Muslims and Christians, respectively. With respect to caste, OBC (Other Backward Castes) constituted 42.9% of the participants, general caste comprised 21.3%, scheduled castes 21.2% and scheduled tribes 9.1%. A majority of the participants were residents of rural areas (66.1%). By household wealth, 17.8% belonged to the poorest quintile, 19.1% to the poorer (the next highest quintile), 20.5% were in the middle quintile, 21.2% in the richer quintile, and 21.3% were from the richest quintile. Health insurance coverage was available to 34.4% of the participants – 24.2% were covered by either one or more government-sponsored or administrated health insurance schemes, and 9.7% possessed one or more non-government health insurance policies. Both government and non-government health insurance cover was available to 0.5% of the participants. Responses were not available from 85.1% for the question on respondents’ occupation, and less than 15% responded to the question on their partner’s education (Table 1).

**Table 1:** Socio-demographic characteristics of participants (women aged 30–49 years)

**Characteristics**	**Sample Frequency**	**Unweighted Proportion**	**Weighted Proportion (95% CI)**
**Age Group (in years)**			
30–34	99,084	27.7	27.5 (27.3–27.7)
35–39	96,074	26.9	26.7 (26.5–26.9)
40–44	79,775	22.3	22.4 (22.2–22.6)
45–49	82,420	23.1	23.4 (23.2–23.6)
**Highest Education**			
No Education	130,054	36.4	35.4 (35.1–35.7)
Primary	55,241	15.5	15.5 (15.3–15.7)
Secondary	139,755	39.1	38.7 (38.4–39.0)
Higher	32,303	9.0	10.4 (10.2–10.7)
**Marital Status**			
Never Married	7416	2.1	1.56 (1.50–1.62)
Formerly Married	26,014	7.3	7.4 (7.3–7.6)
Currently Married	323,923	90.6	91.0 (90.9–91.2)
**Religion**			
Hindu	271,320	75.9	82.3 (81.9–82.7)
Muslim	40,352	11.3	12.1 (11.8–12.5)
Christian	26,913	7.5	2.6 (2.5–2.7)
Sikh	8674	2.4	1.7 (1.6–1.8)
Buddhist/ Neo Buddhist	4886	1.4	0.67 (0.60–0.74)
Other	5208	1.5	0.62 (0.56–0.69)
**Caste**			
Scheduled Caste	66,434	18.6	21.2 (20.8–21.6)
Scheduled Tribe	66,777	18.7	9.1 (8.8–9.3)
OBC	136,093	38.1	42.9 (42.5–43.3)
General	69,003	19.3	21.3 (21.0–21.7)
Don’t Know/ Not Recorded	19,046	5.3	5.5 (5.3–5.7)
**Residence**			
Urban	92,574	25.9	33.9 (33.6–34.3)
Rural	264,779	74.1	66.1 (65.7–66.4)
**Household Wealth Index**			
Poorest	72,074	20.2	17.8 (17.6–18.1)
Poorer	76,424	21.4	19.1 (18.9–19.4)
Middle	74,540	20.9	20.5 (20.3–20.8)
Richer	69,800	19.5	21.2 (20.9–21.5)
Richest	64,515	18.0	21.3 (21.0–21.7)
**Health Insurance**			
Not Covered	227,413	63.6	65.6 (65.3–66.0)
Govt Schemes	83,469	23.4	23.4 (23.1–23.7)
Non-Govt Schemes	42,793	12.0	9.7 (9.5–9.8)
More than 1 Govt Scheme	2143	0.60	0.78 (0.73–0.84)
More than 1 Non-Govt Scheme	67	0.02	0.02 (0.01–0.03)
Both Govt & Non-Govt Schemes	1468	0.4	0.52 (0.47–0.57)
**Occupation**^**a**^			
Unemployed	31,628	8.8	9.0 (8.8–9.3)
Employed	22,024	6.2	5.9 (5.7–6.1)
Don’t Know/ Not Recorded	303,701	85.0	85.1 (84.7–85.5)
**Partner's Education**^**a**^			
No Education	11,382	3.2	3.1 (3.0–3.2)
Primary	8080	2.3	2.3 (2.2–2.4)
Secondary	26,534	7.4	7.2 (7.0–7.5)
Higher	6476	1.8	2.0 (1.9–2.1)
Don't Know/ Not Recorded	304,881	85.3	85.4 (85.0–85.7)
**Age at First Sex**			
< 18 years	137,226	38.4	41.6 (41.3–41.9)
> = 18 years or Not had Sex	206,250	57.7	54.9 (54.6–55.2)
Don’t Know	13,877	3.9	3.45 (3.37–3.55)
**Parity**			
0–2 childbirth	179,673	50.3	52.5 (52.2–52.8)
> 2 childbirth	177,680	49.7	47.5 (47.2–47.8)
**IUD**			
No	330,305	92.4	93.6 (93.4–93.7)
Yes	20,514	5.8	5.0 (4.9–5.2)
Not Recorded	6534	1.8	1.4 (1.3–1.5)
**Pill-OCP**			
No	297,650	83.3	85.0 (84.8–85.3)
Yes	53,169	14.9	13.5 (13.3–13.8)
Not Recorded	6534	1.8	1.41 (1.35–1.47)
**Tobacco use**			
No	324,051	90.7	93.8 (93.6–93.9)
Yes	33,302	9.3	6.2 (6.1–6.4)
**H/o STI**^**a**^			
No	51,144	14.3	14.2 (13.9–14.6)
Yes	2546	0.7	0.68 (0.63–0.74)
Don’t Know/ Not Recorded	303,663	85.0	85.1 (84.7–85.5)

^a^85% of the responses are “*Don’t Know/Not Recorded*”

Age at first sexual activity was less than 18 years of completed age for 41.6% of the participants. 47.5% of the participants had given birth more than twice. IUDs were used by 5.0% of participants as the method of contraception, while 13.5% have used OCPs. Tobacco use was present among 6.2% of the participants. Only 15% of respondents answered the question related to history of STI – 0.7% have had STIs in the past. Since the response rate for participant’s occupation, partner’s education and history of STI were very low, these three were not included for calculating the adjusted odds of prevalence for cervical cancer screening. Also, the response “Don’t know/Not recorded” for IUD use, OCP use and age at first sexual activity were not included while calculating the adjusted odds, in order to avoid collinearity.

### Prevalence of screening

The following observations are derived from Table 2. The prevalence of screening significantly increases with increasing age. The adjusted odds of screening for cervical cancer increase with increasing age – they are 1.21, 1.46 and 1.64 for the age groups 35–39, 40–44 and 45–49 years, respectively, with reference to the 30–34 years age group. The prevalence of screening for cervical cancer is significantly higher among women with some level of education (primary, secondary or tertiary) when compared to women with no education. The adjusted odds of having been screened for cervical cancer are 1.42, 1.44 and 1.51 for women with primary, secondary and higher education respectively, when compared to women with no education. Christian women have significantly higher adjusted odds (1.82) for having undergone screening for cervical cancer, while Muslim women have significantly lower adjusted odds (0.73) for having undergone screening for cervical cancer in comparison to Hindu women. With respect to caste and with scheduled caste women as reference, the adjusted odds are significantly lower for women from scheduled tribes and the general category (0.45, and 0.56 respectively), while those of women from other backward castes are not significantly different. The marital status of the women and the type of place of residence do not show any association with the prevalence of screening for cervical cancer. Among the women with different household wealth, the odds (adjusted) of having undergone screening for cervical cancer are significantly higher among the wealthier quintiles—poorest (reference, 1.0), poorer (1.32), middle (1.59), richer (1.54) and richest (1.55). The availability of government health insurance coverage is associated with significantly higher prevalence of cervical cancer screening, either with a single scheme (adjusted odds 1.22) or multiple schemes (adjusted odds 2.08). Non-government health insurance schemes are associated with significantly lower prevalence of cervical cancer screening (adjusted odds 0.53).

**Table 2:** Prevalence of cervical cancer screening

**Characteristics**	**Weighted Prevalence in %**	**Adjusted Odds Ratio (95% CI)**	***P* Value**
**Age Group (in 5 years)**			
30–34	1.6 (1.4–1.7)	Ref	
35–39	1.9 (1.7–2.0)	1.21 (1.08–1.35)	0.001
40–44	2.1 (2.0–2.4)	1.46 (1.30–1.65)	< 0.001
45–49	2.4 (2.2–2.5)	1.64 (1.46–1.83)	< 0.001
**Highest Education**			
No Education	1.4 (1.3–1.5)	Ref	
Primary	2.1 (1.9–2.3)	1.43 (1.26–1.61)	< 0.001
Secondary	2.3 (2.1–2.5)	1.44 (1.29–1.62)	< 0.001
Higher	2.4 (2.1–2.8)	1.51 (1.27–1.79)	< 0.001
**Marital Status**			
Never Married	0.9 (0.6–1.3)	Ref	
Formerly Married	2.1 (1.8–2.4)	0.98 (0.38–2.51)	0.962
Currently Married	2.0 (1.9–2.1)	1.01 (0.40–2.55)	0.987
**Religion**			
Hindu	2.0 (1.9–2.1)	Ref	
Muslim	1.2 (1.0–1.3)	0.73 (0.62–0.86)	< 0.001
Christian	3.8 (3.1–4.5)	1.82 (1.48–2.24)	< 0.001
Sikh	2.5 (1.7–3.6)	1.13 (0.77–1.66)	0.545
Buddhist/ Neo Buddhist	2.7 (1.7–4.2)	1.19 (0.73–1.95)	0.477
Other	2.7 (0.9–7.4)	2.17 (0.74–6.30)	0.156
**Caste**			
Schedule Caste	2.3 (2.1–2.7)	Ref	
Schedule Tribe	0.9 (0.8–1.1)	0.45 (0.35–0.57)	< 0.001
OBC	2.3 (2.2–2.5)	0.92 (0.80–1.06)	0.260
General	1.5 (1.4–1.7)	0.56 (0.47–0.66)	< 0.001
Don’t Know/ Not Recorded	0.9 (0.6–1.3)	0.42 (0.30–0.59)	< 0.001
**Place of Residence**			
Urban	2.3 (2.1–2.6)	Ref	
Rural	1.8 (1.6–1.9)	0.91 (0.78–1.07)	0.254
**Household Wealth Index**			
Poorest	0.99 (0.88–1.1)	Ref	
Poorer	1.6 (1.5–1.8)	1.32 (1.13–1.54)	0.001
Middle	2.2 (2.0–2.5)	1.59 (1.36–1.85)	< 0.001
Richer	2.4 (2.2–2.6)	1.54 (1.30–1.82)	< 0.001
Richest	2.4 (2.2–2.7)	1.55 (1.27–1.89)	< 0.001
**Health Insurance**			
Not Covered	1.9 (1.7–2.0)	Ref	
Govt Schemes	2.5 (2.3–2.8)	1.22 (1.10–1.36)	< 0.001
Non-Govt Schemes	1.0 (0.8–1.1)	0.53 (0.44–0.64)	< 0.001
More than 1 Govt Scheme	4.4 (3.3–5.9)	2.08 (1.52–2.83)	< 0.001
More than 1 Non-Govt Scheme	1.7 (0.2–11.0)	0.87 (0.12–6.37)	0.892
Both Govt & Non-Govt Schemes	3.2 (2.2–4.8)	1.49 (0.97–2.28)	0.067
**Occupation**			
Not working	1.7 (1.5–2.0)	Not included in the model^a^	
Working	2.4 (2.0–2.9)		
Don’t Know/Not Recorded	2.0 (1.8–2.1)		
**Partner’s Education**			
No Education	1.9 (1.5–2.3)	Not included in the model^a^	
Primary	2.2 (1.7–2.8)		
Secondary	2.0 (1.7–2.3)		
Higher	2.4 (1.8–3.1)		
Don’t Know/ Not Recorded	2.0 (1.8–2.1)		
**Age at First Sex**			
< 18 years	1.8 (1.7–1.9)	Ref	
> = 18 years or Not had Sex	2.1 (2.0–2.3)	1.00 (0.91–1.09)	0.959
Don’t Know	2.0 (1.7–2.4)	Not included in the model^b^	
**Parity**			
0–2 childbirth	2.3 (2.1–2.5)	Ref	
> 2 childbirth	1.6 (1.5–1.7)	0.78 (0.71–0.86)	< 0.001
**IUD**			
No	1.9 (1.8–2.1)	Ref	
Yes	2.6 (2.2–3.1)	1.20 (0.99–1.45)	0.066
Not Recorded	0.8 (0.5–1.2)	Not included in the model^b^	
**Pill-OCP**			
No	2.2 (2.0–2.3)	Ref	
Yes	0.8 (0.7–0.9)	0.42 (0.36–0.49)	< 0.001
Not Recorded	0.8 (0.5–1.2)	Not included in the model^b^	
**Tobacco use**			
No	2.0 (1.9–2.2)	Ref	
Yes	1.1 (0.9–1.3)	0.75 (0.62–0.90)	0.002
**H/o STI**			
No	2.1 (1.8–2.4)	Not included in the model^a^	
Yes	0.8 (0.4–1.4)		
Don’t Know/ Not Recorded	2.0 (1.8–2.1)		

^a^85% of responses are “*Don’t know / Not recorded*”

^b^Excluded due to collinearity

Analysis of the prevalence of screening among women with risk factors for cervical cancer shows that high parity (more than two births), oral contraceptive pill usage and tobacco use significantly influence the odds of screening negatively and are associated with lower prevalence of screening. Age at first sexual activity and intrauterine contraceptive device use are not significant factors in determining the prevalence of cervical cancer screening.

The prevalence pattern of cervical cancer screening among different types of health insurance schemes is presented in Table 3. Women with state government–sponsored health insurance schemes have significantly higher prevalence of cervical cancer screening with odds of 1.57 when compared to women without any health insurance coverage. Participants covered under more than one government insurance scheme have significantly higher screening prevalence (2.44). Women covered only under Rashtriya Swasthya Bima Yojana Scheme or certain non-government health insurance schemes have significantly lower screening prevalence.

**Table 3:** Cervical cancer screening among Health insurance coverage

**Characteristics**		**Sample**			**Cervical Cancer Screening**		
		**Frequency**	**Unweighted Proportion**	**Weighted Proportion (95%CI)**	**Weighted Prevalence in %**	**Odds Ratio (95% CI)**	***P* Value**
	**No**	**227,413**	**63.6**	**65.6 (65.3–66.0)**	**1.9**	**Ref**	
Govt Schemes	ESIS	2418	0.68	0.76 (0.70–0.82)	2.7	1.46 (0.95–2.25)	0.081
	CGHS	6746	1.89	1.74 (1.66–1.82)	2.2	1.19 (0.86–1.64)	0.296
	State HI	52,437	14.7	15.6 (15.3–15.8)	2.9	1.57 (1.39–1.76)	< 0.001
	RSBY	21,868	6.12	5.32 (5.17–5.46)	1.4	0.77 (0.63–0.93)	0.008
Non Govt Schemes	Community HI	335	0.09	0.12 (0.10–0.14)	4.1	2.25 (0.76–6.67)	0.142
	Employer HI	607	0.17	0.23 (0.20–0.27)	2.5	1.35 (0.69–2.62)	0.383
	Employer Reimbursement	523	0.15	0.18 (0.16–0.22)	3.1	1.67 (0.67–4.14)	0.277
	Private HI	2184	0.61	0.77 (0.71–0.83)	1.7	0.90 (0.60–1.35)	0.607
	Other HI	39,144	10.95	8.35 (8.20–8.51)	0.8	0.40 (0.32–0.50)	< 0.001
Covered under more than 1 Govt Scheme		2143	0.60	0.78 (0.73–0.84)	4.4	2.44 (1.80–3.29)	< 0.001
Covered under more than 1 Non-Govt Scheme		67	0.02	0.02 (0.01–0.03)	1.7	0.88 (0.12–6.47)	0.900
Covered under both Govt & Non-Govt Schemes		1468	0.41	0.52 (0.47–0.57)	3.2	1.75 (1.16–2.64)	0.008

The nationwide average prevalence of screening for cervical cancer among women in the age group 30–49 years is 1.97% (1.85–2.09%, 95% C.I). Screening prevalence is comparatively higher in the Southern region (5.0) than the other regions – West (1.7), Central (1.2), North (0.9), North-East (0.6) and the East (0.6) (Table 4).

**Table 4:** Region-wise prevalence of cervical cancer screening

**Region**	**Sample**			**Cervical Cancer Screening**		
	**Frequency**	**Unweighted Proportion**	**Weighted Proportion (95%CI)**	**Weighted Prevalence in %**	**Odds Ratio (95% CI)**	***P* Value**
North	65,687	18.4	13.1 (12.9–13.2)	0.9 (0.8–1.1)	Ref	
Central	82,520	23.1	23.2 (22.9–23.4)	1.2 (1.1–1.4)	1.35 (1.09–1.67)	0.006
North East	52,928	14.8	3.8 (3.7–3.9)	0.6 (0.5–0.7)	0.60 (0.47 -0.77)	< 0.001
West	37,811	10.6	14.8 (14.6–15.1)	1.7 (1.4–2.1)	1.86 (1.40–2.48)	< 0.001
East	55,598	15.5	21.9 (21.7–22.2)	0.6 (0.5–0.7)	0.60 (0.47–0.76)	< 0.001
South	62,809	17.6	23.2 (22.9–23.5)	5.0 (4.6–5.4)	5.62 (4.62–6.84)	< 0.001
India	357,353	-	-	1.97 (1.8–2.1)	-	-

States are grouped into six regions based on their geographic location, as mentioned in the States Reorganisation Act, 1956

Northern region—Chandigarh, Delhi, Haryana, Himachal Pradesh, Jammu and Kashmir, Ladakh, Punjab, and Rajasthan

North East—Assam, Arunachal Pradesh, Manipur, Meghalaya, Mizoram, Nagaland, Tripura and Sikkim

Central region—Chhattisgarh, Madhya Pradesh, Uttarakhand and Uttar Pradesh

East region—Bihar, Jharkhand, Odisha, and West Bengal

West region—Dadra and Nagar Haveli and Daman and Diu, Goa, Gujarat, and Maharashtra

South region—Andhra Pradesh, Karnataka, Kerala, Puducherry, Tamil Nadu, Telangana, Andaman and Nicobar Islands and Lakshadweep

**Fig. 1:**
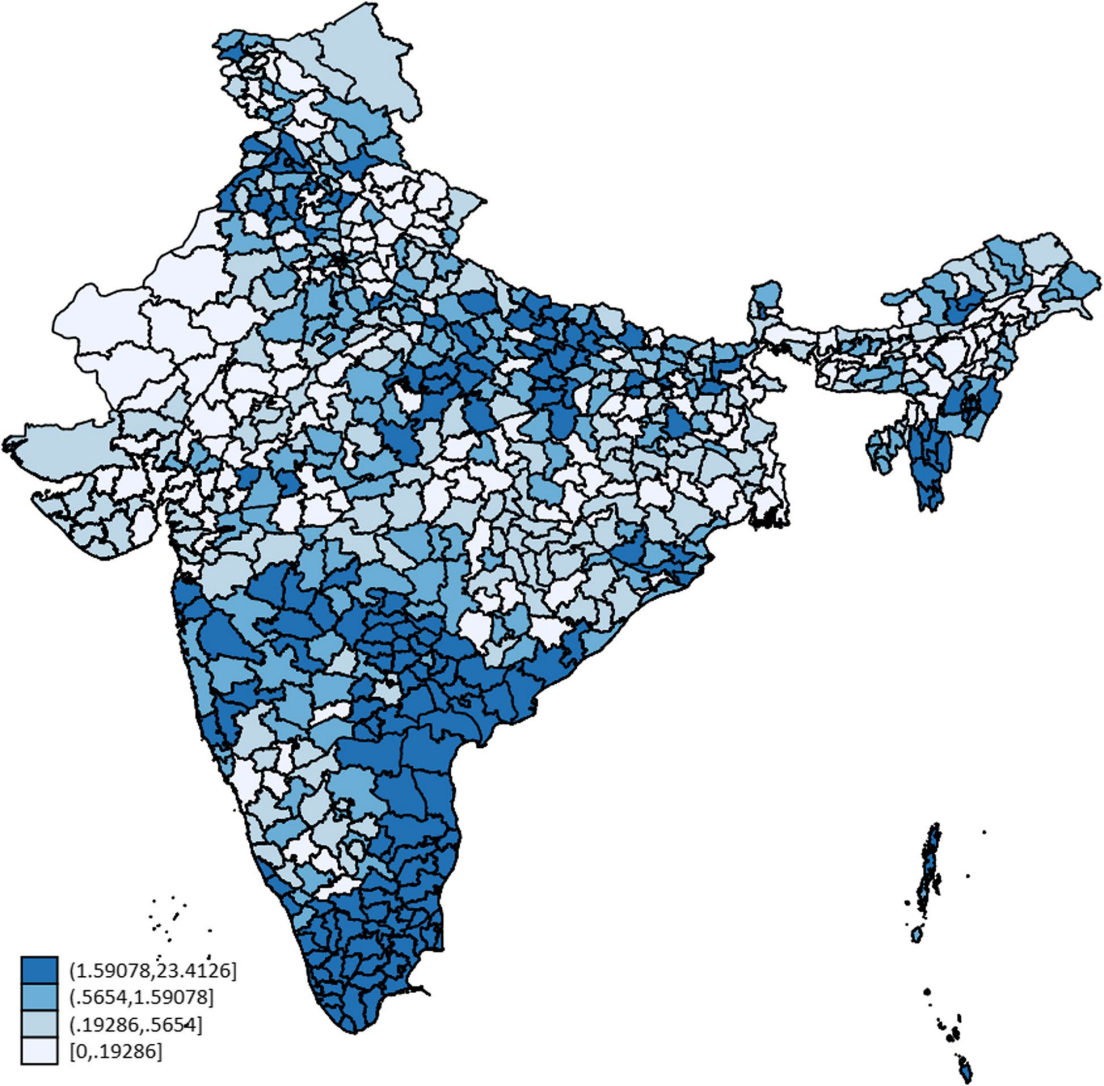
Cervical cancer screening: district-wise prevalence (%) among women aged 30–49 years, India, NFHS-5^+^. ^+^ Map is not representative of the borders of the Country. Areas that were not included in the DHS survey are not shown in the map

The prevalence ranges widely between the states, from the lowest (0.2%) in West Bengal and Assam to the highest (10.1%) in Tamil Nadu. The state-wise prevalence among women aged 30–49 years is presented in Table 5. Tamil Nadu, Puducherry, Mizoram, Andhra Pradesh, Kerala, Telangana, Punjab, Maharashtra, Andaman & Nicobar and Manipur have higher prevalence of screening than the national average. The district-wise prevalence of cervical cancer screening is depicted in the Fig. 1.

**Table 5:** Cervical cancer screening. State / Union Territory-wise weighted prevalence among women aged 30–49 years

**State**	**Sample, Frequency**	**CaCx screening Weighted Prevalence**	**95% C. I**
**Tamil Nādu**	14,655	10.1	8.9–11.3
**Puducherry**	2120	7.6	4.9–11.5
**Mizoram**	4029	7.0	5.4–9.1
**Andhra Pradesh**	6171	4.7	4.0–5.6
**Kerala**	6631	3.5	3.1–4.1
**Telangana**	14,930	3.4	3.0–3.9
**Punjab**	11,571	2.6	1.9–3.5
**Maharashtra**	17,923	2.5	1.9–3.1
**Andaman & Nicobar Islands**	1401	2.4	1.7–3.6
**Manipur**	4390	2.2	1.6–2.8
**Lakshadweep**	680	1.7	0.9–3.1
**Chandigarh**	370	1.6	0.4–6.5
**Uttar Pradesh**	39,893	1.6	1.4–1.8
**Goa**	1169	1.2	0.7–2.0
**Odisha**	14,460	0.9	0.7–1.2
**Himachal Pradesh**	6090	0.9	0.6–1.4
**Madhya Pradesh**	22,546	0.8	0.6–1.1
**Arunachal Pradesh**	10,282	0.8	0.6–1.1
**Bihar**	18,013	0.8	0.7–1.1
**Haryana**	10,831	0.8	0.6–1.0
**NCT of Delhi**	5457	0.7	0.5–1.0
**Tripura**	3919	0.7	0.4–1.0
**Meghalaya**	5935	0.6	0.3–1.2
**Sikkim**	1823	0.6	0.2–1.6
**Karnataka**	16,221	0.5	0.4–0.8
**Jammu & Kashmir**	10,787	0.5	0.3–0.7
**Jharkhand**	12,245	0.5	0.3–0.6
**Uttarakhand**	6505	0.4	0.2–0.8
**Dadra, Nagar Haveli and Daman & Diu**	1330	0.4	0.1–1.4
**Rajasthan**	19,416	0.4	0.3–0.6
**Chhattisgarh**	13,576	0.3	0.2–0.4
**Ladakh**	1165	0.3	0.1–0.9
**Nagaland**	5005	0.3	0.2–0.5
**Gujarat**	17,389	0.2	0.2–0.4
**Assam**	17,545	0.2	0.1–0.3
**West Bengal**	10,880	0.2	0.1–0.3
**India**	**357,353**	**1.97**	**1.8–2.1**

### Wealth-based inequality

**Fig. 2:**
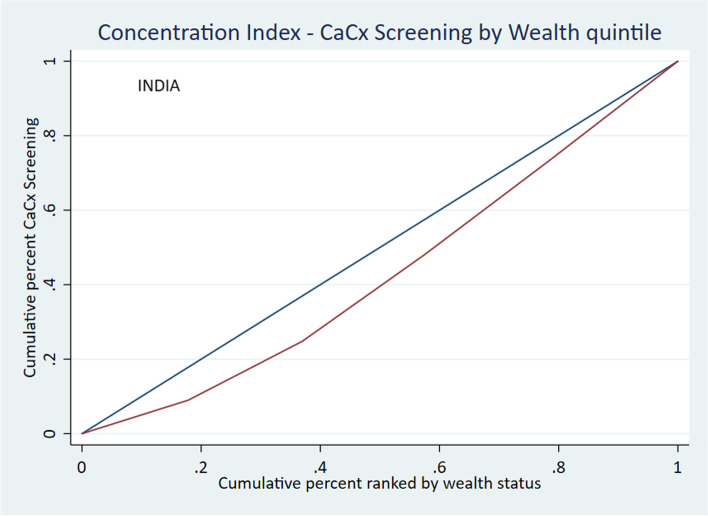
Wealth-based inequality in cervical cancer screening. Concentration Index

**Fig. 3:**
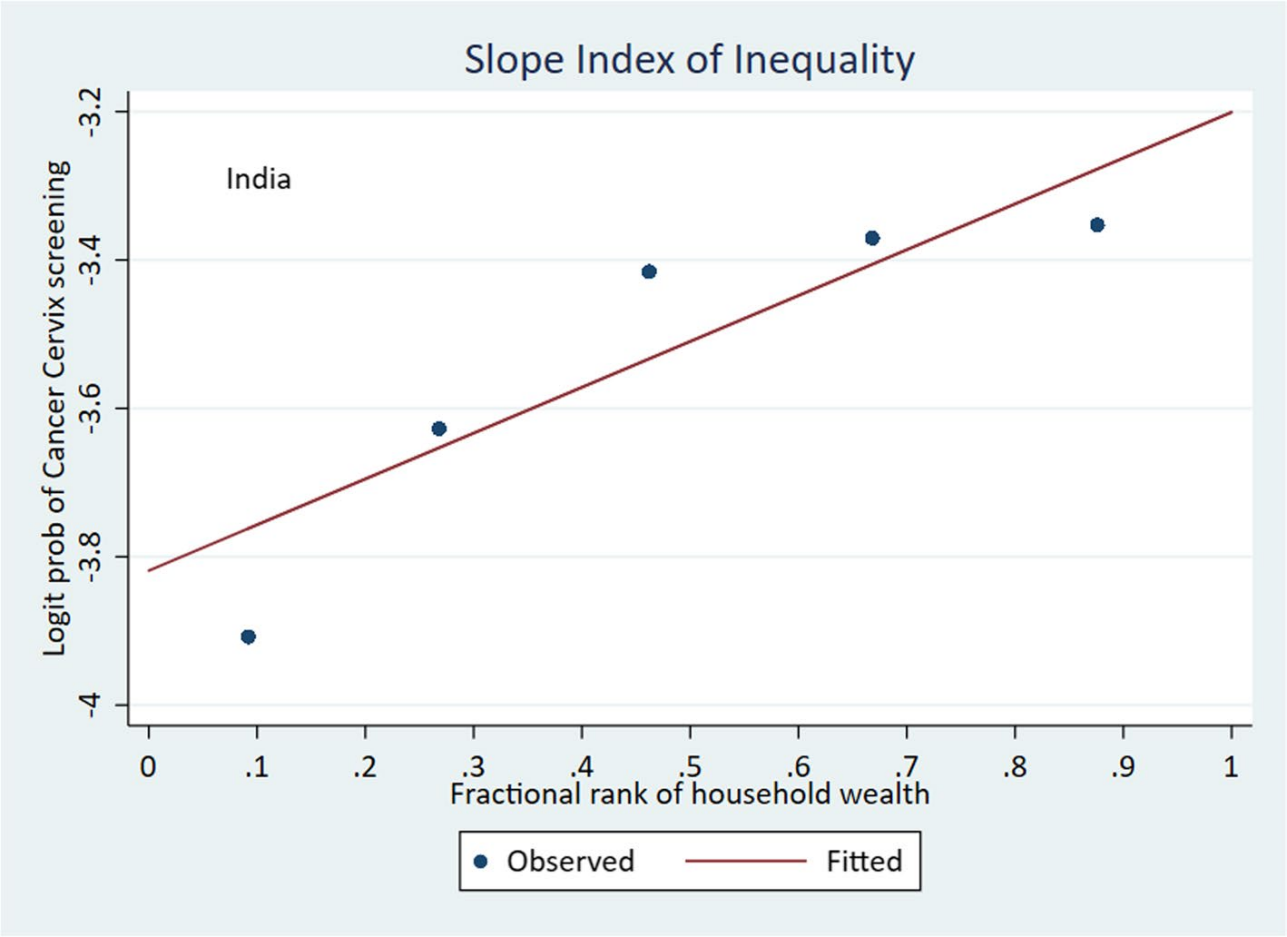
Wealth-based inequality in cervical cancer screening. Slope Index of Inequality

The Relative Concentration Index (RCI), Corrected Concentration Index (CCI) and the Slope Index of Inequality (SII) are measures used to assess the level of inequality in the prevalence of cervical cancer screening with respect to the wealth quintiles – poorest, poorer, middle, richer and richest. A negative value for an index indicates that the concentration of the health variable is among the poor, and a positive value indicates that the concentration of the health variable is among the richer groups. The results of the analysis show pro-rich wealth-based inequality at the national level, as indicated by the significantly positive values of all three indexing measures of inequality (Table 6). The concentration curve lies below the line of equality, indicating a significantly higher concentration of screening prevalence among women from wealthier quintiles (Fig. 2). The graph for Slope Index of Inequality shows the line rising from left to right, a positive slope, which indicates higher prevalence of cervical cancer screening among the wealthier quintiles (Fig. 3).

**Table 6:** Wealth-based inequality in cervical cancer screening. Measures of inequality

	**Measure**	**Co. Eff**	**S.E**	**95% C.I**	***P***
**India**	RCI	0.22	0.001	0.20–0.24	< 0.001
	CCI	0.02	0.001	0.01–0.02	< 0.001
	SII	0.018	0.001	0.015–0.020	< 0.001

**Fig. 4:**
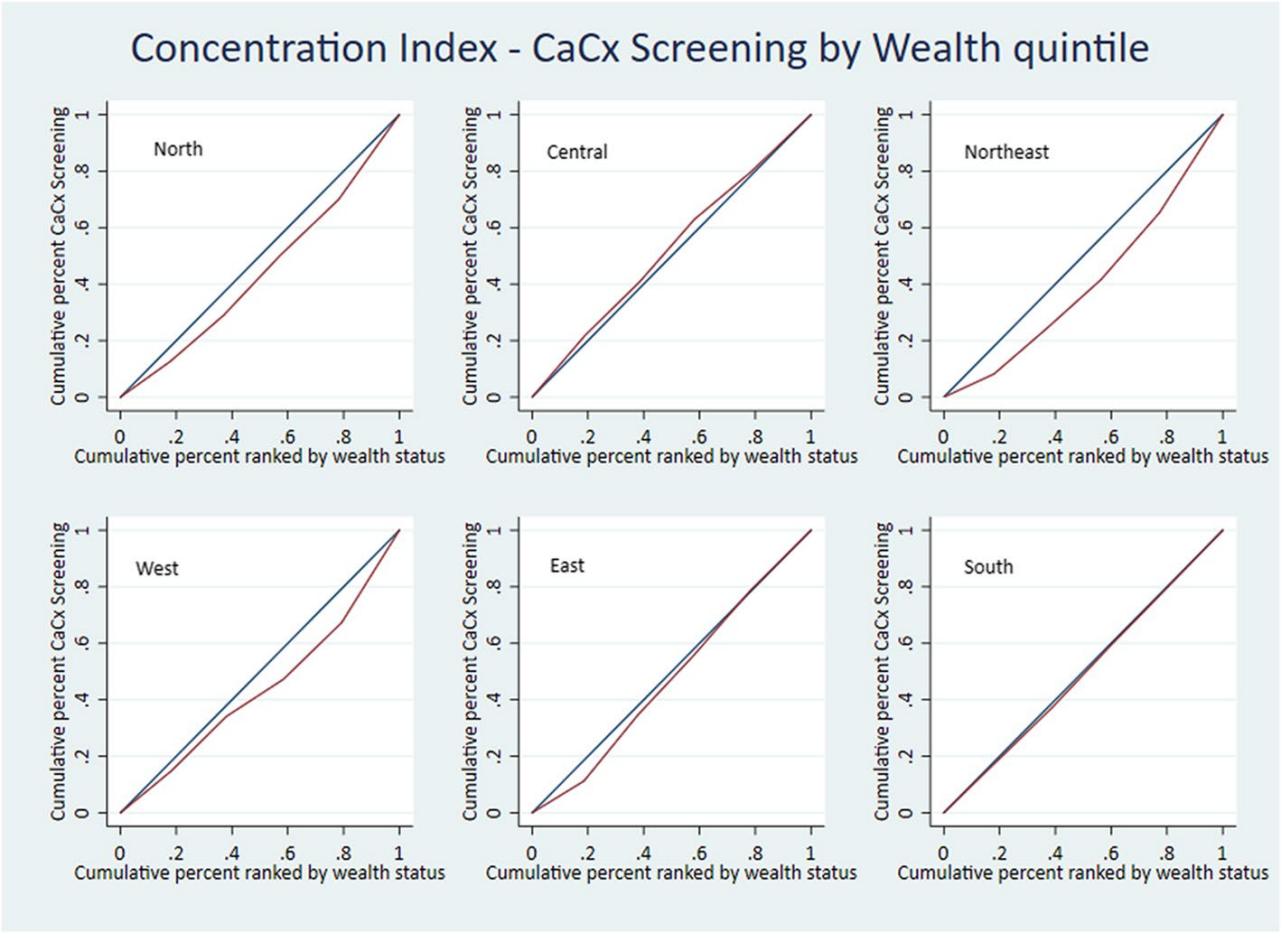
Region-wise – Wealth-based inequality in cervical cancer screening. Concentration curve

**Fig. 5:**
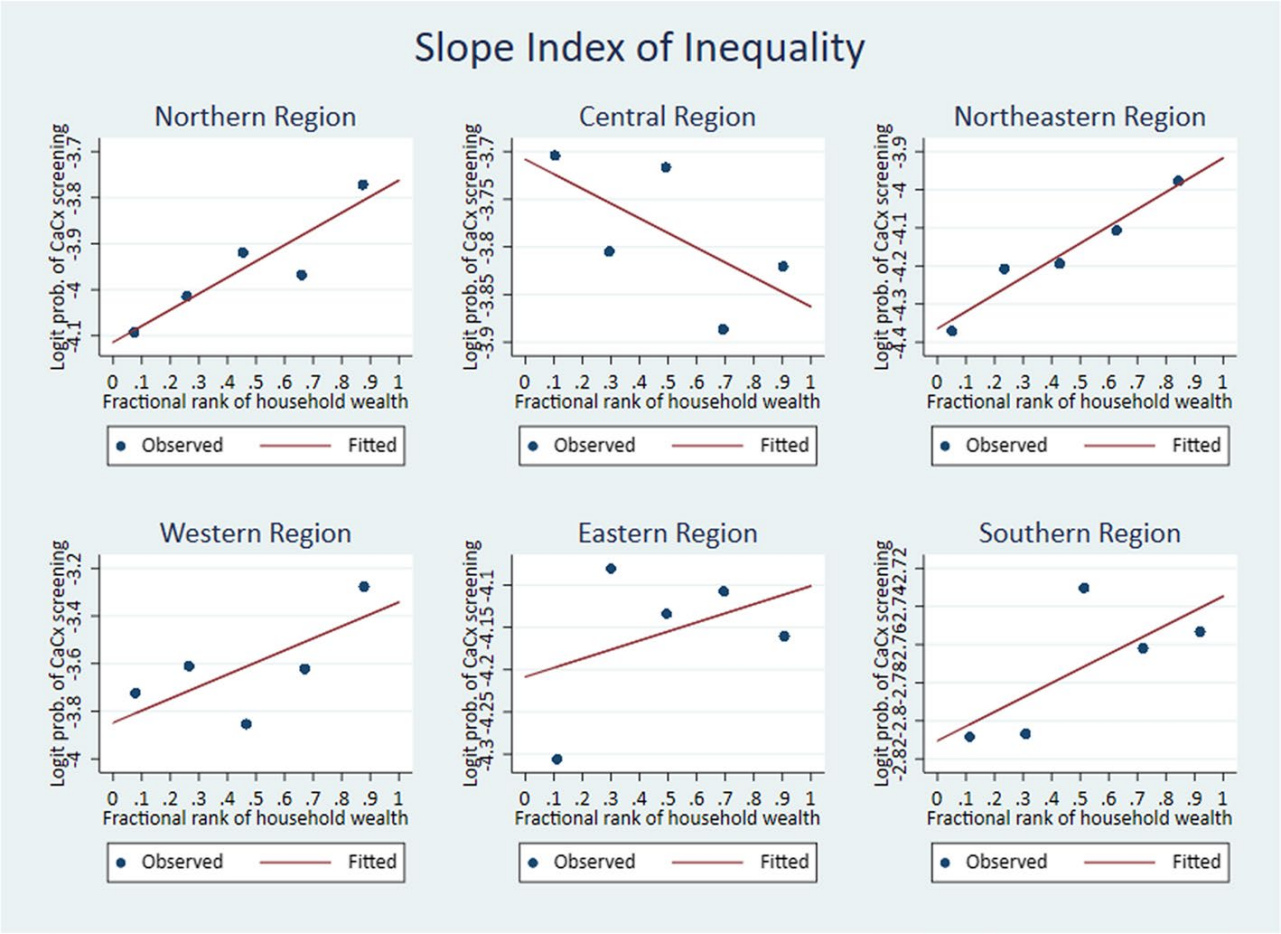
Region-wise – Wealth-based inequality in cervical cancer screening. Slope Index of Inequality

At the regional level, the West and North-East regions show significantly positive values of RCI, CCI and SII, indicating significantly higher prevalence of cervical cancer screening among the richer quintiles. Analysis shows significantly positive values for RCI & CCI for the South, while the Northern region returns a significantly positive value for SII only. These are also indicative of a higher concentration of cervical cancer screening prevalence among wealthier women. The values for the Eastern region are insignificant and hence inconclusive. The Central region shows significant negative values for the RCI, CCI and SII, indicating a significantly higher concentration of cervical cancer screening among the women belonging to poor quintiles (Table 7). The concentration curves for the five regions North, North-East, West, East and South are below the line of equality, implying significantly higher prevalence of cervical cancer screening among wealthier quintiles. The Central region’s concentration curve is above the line of equality, meaning cervical cancer screening prevalence is significantly higher among the poor (Fig. 4). In line with the concentration curves, the SII curves also show significantly higher prevalence of cervical cancer screening among wealthier women in the North, North-East, West, East and South regions and women belonging to poor quintiles in the Central region (Fig. 5).

**Table 7:** Region-wise – Wealth-based inequality in cervical cancer screening

**Region**	**Measure**	**Co. Eff**	**S.E**	**95% C. I**	***P***
**North**	RCI	0.05	0.03	(-0.01)-0.11	0.083
	CCI	0.002	0.001	(-0.0003)-0.004	0.089
	SII	0.01	0.002	0.003–0.009	< 0.001
**Central**	RCI	-0.05	0.02	(-0.9)- (-0.002)	0.04
	CCI	-0.002	0.001	(-0.005)- (0.0001)	0.04
	SII	-0.003	0.002	(-0.007)- (-0.000)	0.049
**Northeast**	RCI	0.1	0.04	0.03–0.17	0.006
	CCI	0.002	0.0009	0.0006–0.004	0.009
	SII	0.01	0.001	0.004–0.009	< 0.001
**West**	RCI	0.21	0.04	0.13–0.29	< 0.001
	CCI	0.01	0.003	0.008–0.021	< 0.001
	SII	0.01	0.005	0.004–0.023	0.005
**East**	RCI	0.03	0.04	(-0.04)-0.10	0.396
	CCI	0.001	0.0008	(-0.0009)-0.002	0.395
	SII	0.002	0.001	(-0.001)-0.004	0.18
**South**	RCI	0.05	0.01	0.03–0.08	< 0.001
	CCI	0.01	0.003	0.005–0.02	< 0.001
	SII	0.0042	0.0039	(-0.004)–0.012	0.294

**Fig. 6:**
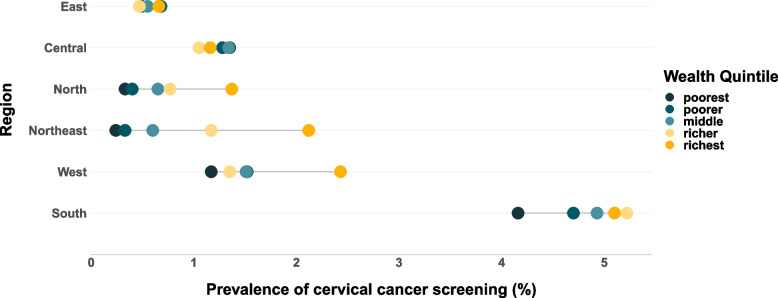
Region-wise – Wealth-based inequality in cervical cancer screening

An equiplot is a graphical way to present the patterns of inequality among different groups within a population. The equiplot in Fig. 6, built based on the data in Table 8, presents the relative prevalence of cervical cancer screening among the wealth quintiles of the six sub-national regions based on the inequality patterns “top, linear and bottom” described by Victora CG et al. and Barros AJ et al. [15, 16]. The colored dots depict the prevalence of cervical cancer screening in the wealth quintiles. In the eastern region the dots are closely packed – the poor and rich are intertwined without any distinctive pattern of hierarchical order of prevalence, indicating an absence of significant inequality in cervical cancer screening based on household wealth. In the central region the darker dots depicting the poor quintiles are on the right side, showing higher prevalence of cervical cancer screening among the poor than the rich, a pattern of pro-poor inequality. The North, North-East and Western regions show a “top-inequality pattern” with the richest quintile placed way ahead of the rest, which is typical of regions with overall lower prevalence where the richest alone manage to avail screening. The southern region shows a “bottom-inequality pattern” with the poorest quintile alone lagging behind all others, typical of a region which is actually beginning to gain acceleration in overall prevalence. This shows that although the poorest quintile of the southern region is placed ahead of the richest quintiles of other regions and the overall prevalence is improving in the southern region, the poorest sections of the southern region are not progressing at the same pace and are left behind compared to other quintiles.

**Table 8:** Prevalence of cervical cancer screening (%) in wealth quintiles across regions

**Region**	**Poorest**	**Poorer**	**Middle**	**Richer**	**Richest**
**North**	0.3	0.4	0.7	0.8	1.4
**Central**	1.3	1.3	1.3	1.0	1.2
**Northeast**	0.2	0.3	0.6	1.2	2.1
**West**	1.2	1.5	1.5	1.4	2.4
**East**	0.5	0.7	0.5	0.5	0.7
**South**	4.2	4.7	4.9	5.2	5.1
**India—Nationwide**	0.99	1.62	2.22	2.37	2.43

## Discussion

The ideal way to avoid morbidity and mortality due to cervical cancer is by preventing the acquisition / chronic persistence of the causative agent, the Human Papilloma Virus in the uterine cervix. This can be achieved by HPV immunization at the appropriate age before the initiation of sexual activity, i.e., risk of exposure to HPV. This prevents risk for the development of squamous cell carcinoma of the cervix. Even though cervical cancer can be completely cured without further recurrences when diagnosed in its initial stages, surgical treatment of CIN has been associated with an increased risk of preterm delivery, lower birth weight, preterm premature rupture of membrane and obstetrical outcomes, especially following “large loop excision of transformation zone (LLETZ)” and “cold-knife conization (CKC) procedures on the uterine cervix” [17]. This emphasizes the importance of preventing an HPV infection.

Diagnosis of cervical cancer in its initial stages by screening is the next best way to prevent mortality due to cervical cancer, because the disease is amenable to treatment if diagnosed early. In its initial stages, cervical cancer does not cause any symptoms, and hence affected people do not seek medical attention. Screening for cervical cancer is considered crucial because it enables detection of the disease in its early stages. Unless identified by screening, cervical cancer will be left undiagnosed and the disease will progress unnoticed. The affected person will develop symptoms as the disease progresses to an advanced stage, making it difficult to achieve a complete cure and thereby prevent mortality [4, 5]. Moreover, early detection by screening is the only way to prevent death due to cervical cancer among people who are already exposed to HPV infection and people who are not suitable candidates for receiving HPV vaccination [18]. In addition, screening is important irrespective of having received an HPV vaccination, because strains not included in the vaccine are also oncogenic [1]. Considering the important role of screening in the elimination of cervical cancer, the WHO’s global strategy to accelerate the elimination of cervical cancer targets covering 70% of women with two episodes of screening, with a high-performance screening test by the year 2030, one episode at 35 years of age and another episode at 45 years of age.

Baseline information on key indicators is necessary to devise implementation strategies for achieving the WHO targets and also to measure periodical progress towards achieving the targets. The baseline parameters on the prevalence of cervical cancer screening derived from NFHS-4 data were reported by Van Dyne et al. [19]. The subsequent survey, NFHS-5, used a more specific question for collecting data on prevalence of cervical cancer screening. Consequently, NFHS-5 presents a markedly lower prevalence of cervical cancer screening across India in comparison to NFHS-4 [19, 13]. An independent study conducted in July 2019 among women aged 25–65 years in a South Indian community revealed that 14.3% had a pelvic exam in their lifetime while only 7.1% had undergone cervical cancer screening at least once in their lifetime [20]. This explains the reason for difference in results when the survey question was about cervical examination (NFHS-4) compared to the survey question about screening for cervical cancer (NFHS-5). As discussed by Van Dyne EA et al., women might have reported cervical examinations that were not related to cervical cancer screening as a positive response to the NFHS-4 questionnaire. This could have led to the perception of a higher prevalence of cervical cancer screening in NFHS-4 compared to NFHS-5 [15, 19]. A systematic review of 78 studies published between 1993–2017 (52 of them between 2012–17) revealed that the percentage of women from the general population of India who have participated in cervical cancer screening ranged from 0.7% to 12.2% [21]. This is in line with the results of the NFHS-5’s reporting of 0.2% to 10.1% of screening prevalence between the states. Moreover, the National Noncommunicable Diseases Monitoring Survey, a national representative survey conducted during 2017–18, reported that 2.3% of women aged 30–49 years had undergone screening for cervical cancer by visual inspection with acetic acid, pap smear or HPV test [22]. This is much more in correlation with the NHFS-5’s prevalence rate of 1.97% for cervical cancer screening among women aged 30–49 years than the NFHS-4’s, where it is reported as 29.8% [19]. Hence, the results presented in this study are appropriate as a baseline for devising implementation strategies and also for measuring the progress towards achieving the WHO targets when future rounds of NFHS data or another data from national representative sample become available.

Inequity in the prevalence of cervical cancer screening exists worldwide based on different factors. In Europe, women living in Eastern, Southern and Northern Europe, with a low or intermediate educational level, widowed women or never-married women, and those with low household income were associated with a lower likelihood of having had a cervical smear test in the past three years when compared to women living in Western Europe, women of a high educational level, married women, and those with a higher household income, respectively. In comparison, India’s pattern shows inequality in the prevalence of cervical cancer screening with higher prevalence among educated women and those with a higher household income. The earlier implementation of organized screening programs in Northern and Western European countries compared to the rest of Europe is proposed as the reason for the higher prevalence of screening [23]. In India, the South, West and Central regions have significantly higher prevalence of cervical cancer screening when compared to East, North-East and Northern regions. The National Program for Control of Cancer, Diabetes, Cardiovascular diseases and Stroke (NPCDCS) was launched in the year 2010, implemented in phases, and it covered the entire nation by the year 2017 [24]. A few states in India were already implementing their own state-level cancer control programs and were in an advantageous position to easily adopt the NPCDCS when it was launched. Tamil Nadu implemented the World Bank–supported NCD control program from the year 2008 using the VIA testing method in facility-based opportunistic mode, and later switched to the NPCDCS program [25, 26]. The early implementation of the program could be one of the reasons for the relatively higher prevalence of cervical cancer screening in Tamil Nadu.

Utilization of cervical cancer tests is higher among women who are daily or occasional smokers in Europe [23]. This is in contrast to India, where the prevalence of cervical cancer screening is lower among women using tobacco. In Zimbabwe, religious affiliations and usage of health facilities act as determinants of participation in cervical cancer screening. Women affiliated with Roman Catholic, Protestant, Pentecostal and Apostolic sects were less likely to screen for cervical cancer compared to those in other religions [27]. Our study also shows that religious affiliations are associated with inequity in prevalence of cervical cancer screening. Christian women in India have significantly higher adjusted odds (1.82) for having undergone screening for cervical cancer, while Muslim women have significantly lower adjusted odds (0.73) for having undergone screening for cervical cancer in comparison to Hindu women. In South Africa, Nigeria, Uganda and Peru, women in rural areas were disproportionately affected by distance and the travel costs incurred in assessing the health facilities for cervical cancer screening [28–31]. For women living in the rural areas of these countries, lack of time due to home care commitments, travel costs and personal safety issues on the way to the health facility are mentioned as the barriers in availing cervical cancer screening. Our study does not find any significant difference in the prevalence of cervical cancer screening among women based on the type of place of residence in India (rural vs urban areas).

A study among United States women aged 21–65 years reported that cervical cancer screening rates varied significantly by type of insurance coverage. Compared with women with employer-based insurance or Medicare (aged ≥ 65 years), women with other types of insurance were 2%–4% less likely to receive a Pap test [32]. A systematic review of 29 observational studies conducted in the United States revealed that lack of health insurance coverage or disruptions in health insurance coverage were consistently statistically significantly associated with lesser receipt of cervical cancer prevention services as well as with advanced stages of cancer at diagnosis and worse survival [33]. Our study shows that government-sponsored health insurance schemes, particularly state government–sponsored schemes, are associated with significantly higher prevalence of cervical cancer screening (O.R-1.57, *p *< 0.001). Women covered under more than one government health insurance scheme have even higher prevalence of cervical cancer screening (O.R-2.44, *p* < 0.001). In Tamil Nadu, the state with the highest prevalence of cervical cancer screening among the Indian states, the state-sponsored Chief Minister’s Comprehensive Health Insurance Scheme covers the confirmatory colposcopy test for women tested VIA positive, and also covers cryotherapy and all other treatments for cervical cancer. The relationship between the availability of further diagnostic tests and treatment for cervical cancer under health insurance and the prevalence of cancer screening has to be studied, as it may be a possible cause for the higher prevalence of cervical cancer screening among women covered under these schemes [34].

The significant difference in prevalence among states, religious groups, caste groups and wealth quintiles necessitate the exploration of reasons for the same. Further studies are required to find the reason for the higher prevalence of cervical cancer screening among women covered under state government–sponsored health insurance schemes when compared to women without insurance coverage and women covered under other types of health insurance schemes. Apart from the social, economic and demographic factors discussed in this study, other influences like behavioral and belief patterns need to be studied and addressed to successfully achieve the targeted coverage of cervical cancer screening. It is established that the majority of women who perceived their health status to be poor had taken a Pap smear test compared to those with perceived excellent health status [35]. Similarly, a fear of screening (that the test would be painful) and a fear of the detection of any abnormal pathology, particularly in the absence of support mechanisms, have been proved to be a reason for the low acceptance of screening tests in rural India [36, 37]. Health education campaigns that address specific religious and cultural issues regarding cervical cancer prevention need to be explored for their effectiveness [38, 39]. The validity of providing alternative choices to women, like offering self-sample collection methods for undergoing screening, needs to be studied with respect to women who did not respond to health education and for whom privacy is the major concern [40, 41]. We conclude that demystifying the reasons for the socio-demographic and economic patterns of difference in prevalence presented above hold the key to achieving the target screening levels and preventing deaths due to cervical cancer.

### Strengths of the study

This is the first study about the prevalence of cervical cancer screening based on the NFHS-5 data, which contains specific information on cervical cancer screening. The role of health insurance on cervical cancer screening is explored for the first time from a national representative database, and wealth-based inequality is explored with relation to cervical cancer screening.

### Limitations of the study

The types of tests commonly used for screening (VIA/Pap/HPV), places of screening (primary/secondary/tertiary care facility/mobile health units/home) and types of facility (public or private) are not described in this study. Moreover, the behaviors, beliefs, social and economic levels, and demographic patterns are dynamic and constantly changing. Hence periodical assessment is necessary for the suitable modification of cancer control programs at required time intervals.

## Data Availability

The datasets analyzed during the current study are available in the Demographic Health Survey website, the datasets are downloaded from the website after registration and approval. The website can be accessed from https://dhsprogram.com/data/available-datasets.cfm.

## Abbreviations

CaCx: Cervical Cancer
CCI: Corrected Concentration Index (Erreygers)
C.I: Confidence Interval
CIX: Concentration Index
CKC: Cold-knife conization
Co. Eff: Co-efficient
Govt: Government
HPV: Human Papilloma Virus
HSIL: High grade Squamous Intraepithelial Lesion
IUD: Intra Uterine contraceptive Device
LLETZ: Large loop excision of transformation zone
LSIL: Low grade Squamous Intraepithelial Lesion
NCD: Non-Communicable Diseases
NFHS: National Family Health Survey
NPCDCS: National Program for Control of Cancer, Diabetes, Cardiovascular diseases and Stroke
OBC: Other Backward Castes
OCP: Oral Contraceptive Pills
Pap: Papanicolaou test
RCI: Relative Concentration Index
RSBY: Rashtriya Swasthya Bima Yojana
SII: Slope Index of Inequality
STI: Sexually Transmitted Infections
VIA: Visual Inspection with Acetic acid
VIF: Variable Inflation Factor
WHO: World Health Organization
